# Changes in clinical practice for central venous access cannulation after the introduction of ultrasound studies in the ICU

**DOI:** 10.1186/2197-425X-3-S1-A73

**Published:** 2015-10-01

**Authors:** Ó Martínez González, D Ballesteros, M Á Alonso Fernández, M Chana, B López Matamala, C Martín Parra, R Blancas

**Affiliations:** Critical Care, Hospital Universitario del Tajo, Aranjuez, Spain

## Introduction

The introduction of ultrasound in the ICU has been extended in recent years. Multiple studies have examined the potential benefits of its use for central venous access canulation, although not yet know what changes occur in clinical practice.

## Objectives

Analyze changes in clinical practice before and after train a group of intensivists in the management of ultrasound-guided technich for central venous access cannulation.

## Methods

Prospective cohort study conducted at the Hospital Universitario del Tajo between September 1, 2011 and September 1, 2014. Two groups were created. A Control Group (CG) involving all central catheters access cannulated between 1 September 2011 and 1 September 2012. The following year the ultrasound was entering in the ICU. The second group, ultrasound group (UG), including all central catheters access cannulated between 1 September 2013 and 1 September 2014. Catheter and localization, the use of ultrasound, date of insertion and removal were collected, major bleeding, pneumothorax, and catheter bacteremia. For statistical analysis, SPSS v20.0 for comparison of qualitative variables the χ2 test and Student´s t test was used for quantitative variables. For rate analysis program Epidat 4.1 was used.

## Results

The number of catheters increased significantly from 141 in CG to 181 in UG. Remarkable was the jugular accesses increased from 35 (24.8%) to 67 (37.0%) (Table 1). In the UG 97 (53.6%) of central veins with ultrasound guidance were cannulated. The mean length mantenance were 6.43 days in the CG versus 6.35 days in UG (p = 0.054). There wasn´t significant differences about pneumothorax observed (Table 1). The bacteremia rates were in the CG of 1.14 / 1000 catheter days and the UG 6.27 / 1000 catheter days, with an RR 5.49 (95% CI 0.44 to 20.0).

**Table 1:** Table 1

	Control Group	Ultrasound Group	p
Catheters number(%)	141	181	0,004
Basilic	2(1,4)	1(0,6)	
Cephalic	32(22,7)	22(12,2)	
Femoral	22(15,6)	36(19,9)	
Subclavian	50(35,5)	55(30,4)	
Yugular	35(24,8)	67(37,0)	
Ultrasound guided(%)	11(7,8)	97(53,6)	0,026
Pneumothorax(%)	2(2,4)	3(2,5)	0,667
Bacteremia(%)	1(0,7)	7(3,9)	0,084

## Conclusions

The use of ultrasound guidance to cannulate central vein access in ICU significantly increases the number of implanted catheters, especially in the jugular. A decrease in pneumothorax or major bleeding is observed. An increase of bacteremia rates is observed.

